# Supplementary material for: Prevention and Management of Operating Room Fire: An Interprofessional Operating Room Team Simulation Case
Source: MedEdPORTAL. 2020 Jan 24;16:10871. doi: 10.15766/mep_2374-8265.10871 (PMC7012309; doi:10.15766/mep_2374-8265.10871)
Supplement: Supplementary file 1 — A. Simulation Case Overview.docx B. Teaching Points.docx C. Slide Introduction.pptx D. Surgical History and Physical Exam.docx E. Debriefing Checklist.docx F. Evaluation Form.docx [file mep-16-10871-s001.zip › _Educational_Summary_Report_10871.pdf]

MedEdPORTAL® The AAMC Journal of Teaching and Learning Resources

Original Publication Open Access

Prevention and Management of Operating Room Fire: An Interprofessional Operating Room Team Simulation Case

Christine L. Mai, MD, MS-HPEd*, Praelada Wongsirimeteekul, MD, Emil Petrusa, PhD, Rebecca Minehart, MD, MS-HPEd, Maureen Hemingway, RN, DNP, May Pian-Smith, MD, Ersne Eromo, MD, Roy Phitayakorn, MD, MHPE (Med)

*Corresponding author: cmai1@partners.org

Abstract

Introduction: Operating room (OR) fire can be a devastating and costly event to patients and health care providers. Prevention and effective management of such fires may present difficulties even for experienced OR staff. **Methods:** This simulation involved a 52-year-old man presenting for excisional biopsy of a cervical lymph node to be performed under sedation. Participants were expected to identify and manage both contained and uncontained fires resulting from ignition by electrosurgical cautery. We conducted weekly multidisciplinary simulations in the mock OR at Massachusetts General Hospital. Participants included surgery and anesthesiology residents, certified registered nurse anesthetists, registered nurses, and surgical technicians. Participants were unaware of the scenario content. Each 90-minute session was divided into three parts: an orientation (10 minutes), the case with rapid cycle debriefing (65 minutes), and a final debriefing with course evaluations (15 minutes). Equipment consisted of a simulation OR with general surgery supplies, general anesthesia equipment, a high-fidelity Laerdal SimMan 3G simulator, a code cart, a defibrillator, dry ice for smoke effects, and a projector with a fire image. **Results:** From April to June 2015, 86 participants completed this simulation. Participants reported that the simulation scenario was realistic (80%), was relevant to their clinical practice (93%), changed their practice (82%), and promoted teamwork (80%). **Discussion:** Prevention and management of OR fire require collaboration and prompt coordination between anesthesiologists, surgeons, and nurses. This simulation case scenario was implemented to train multidisciplinary learners in the identification and crisis management of such an event.

Keywords
Simulation, Interprofessional, Operating Room Fire, Fire Triangle, Interprofessional Education

Educational Objectives

By the end of this activity, learners will be able to:

1. Identify situations conducive to operating room (OR) fire.
2. Manage OR fire in terms of RACE (rescue, alert/alarm, contain, extinguish/evacuate).
3. Reduce adverse outcomes associated with OR fires.
4. Apply the core concepts of crisis management.

Introduction

Operating room (OR) fires are rare but potentially catastrophic events that have important consequences to both patients and health care providers. The ECRI Institute estimates that 90-100 surgical fires occur in the OR per year in the US.[1] Adverse patient outcomes associated with OR fires may include major or minor burns, inhalational injuries, infection, and death.[2] Related patient adverse outcomes may include psychological trauma, prolonged hospitalization, delayed or cancelled surgery, additional hospital resource utilization, and liability.[2] For health care providers involved with the experience, psychological and personal safety issues may arise immediately during and months to years after an OR fire. Although every institution is required to train its employees on overall fire safety, few programs emphasize the unique aspects of OR fires or incorporate multidisciplinary team training and simulation education into health care providers' fire training.[3] Specifically, the OR environment contains volatile gases that can act as accelerants in an OR fire and must be stopped through centralized shutoff valves. Similarly, evacuation from a fire in the OR is different from other clinical environments considering that the patient may be under general anesthesia and difficult to transport. In our simulation scenario,

Citation:
Mai CL, Wongsirimeteekul P, Petrusa E, et al. Prevention and management of operating room fire: an interprofessional operating room team simulation case. *MedEdPORTAL*. 2020;16:10871.
https://doi.org/10.15766/mep_2374-8265.10871

Copyright © 2019 Mai et al. This is an open-access publication distributed under the terms of the Creative Commons Attribution license.

we emphasize crisis resource management during sequentially contained and uncontained OR fires to promote interpersonal skills, team dynamics, and practical skills training for such an event.

A team of surgeons, anesthesiologists, and nursing educators developed this simulation to include learning points for each participating profession: general surgery and anesthesiology residents, certified registered nurse anesthetists (CRNAs), OR nurses, and surgical technologists. Even though there may be some institutional resources directed at how to prevent fires, currently there are few available resources on how to manage an OR fire. Although *MedEdPORTAL* has a resource on managing an airway fire due to electrocautery during a tracheostomy,[4] our scenario discusses management of contained versus uncontained OR fire. Although not explicitly defined by standards of the Joint Commission on Accreditation of Healthcare Organizations, a contained fire is typically thought of as a fire that is limited in scope or spread. Alternatively, an uncontained fire is a fire that has not been contained and continues to spread within the OR, on the OR floor, or throughout the hospital. This scenario involves intraoperative fires requiring identification of the factors conducive to initiating a fire and the process of reporting and then managing the event, including OR evacuation.

The case occurs in a simulated OR environment with all associated equipment. In the scenario, a 52-year-old man presents for excisional biopsy of a cervical lymph node to evaluate for lymphadenopathy concerning for lymphoma. He is sedated for the procedure.

Target Audience

The targeted audience includes surgical residents, anesthesia residents, CRNAs, OR nurses, and surgical technicians. The scenario is applicable to all levels of learners.

Methods

Faculty Preparation

Simulation facilitators received all details of the case along with an ideal scenario flow and typical management mistakes (Appendix A). Teaching points were also provided depending on how participants responded to events (Appendix B). The learners did not have any training prerequisites.

Equipment/Environment

The primary piece of equipment was a high-fidelity simulator (SimMan 3G; Laerdal Medical, Stavanger, Norway), which was set up as a patient undergoing monitored anesthesia care with a nasal cannula and one large-bore peripheral IV catheter already placed. The simulator had been surgically prepped and draped. It had a typical hospital identification wristband with the patient's name and medical record number. Vital signs such as pulse oximeter, noninvasive blood pressure, and electrocardiogram set by faculty were monitored in real time with a standard simulated monitor. Audiovisual equipment included two video cameras and several microphones suspended from the ceiling. The instructors wore wireless headsets to communicate with the directors for the simulation session.

Other equipment included standard surgical instruments, blue Weck clip applicators, closing suture, a specimen jar, a disabled electrosurgical unit, and appropriate paperwork. The contained fire was created using dry ice and water in a suction canister, with the smoke vapors piped up via an outlet near the mannequin's neck. The uncontained fire was a projector image of a blazing fire. This in situ simulation took place at Massachusetts General Hospital in an actual decommissioned clinical OR, which had a surgical bed, overhead lights, and cabinets with standard OR supplies; however, the simulation could be replicated in another location outfitted as an OR.

For anesthesia, a functional anesthesia machine with no volatile agents but with oxygen and air via pipeline was placed in the room with standard airway equipment including Mac 3 and Miller 2 blades. Other equipment included endotracheal tubes, oral airways, suction tubing, a self-inflating bag valve mask, and tape. Simulated medications included a variety of prepared syringes: propofol, succinylcholine, midazolam, fentanyl, vecuronium, phenylephrine, ephedrine, atropine, and epinephrine in standard doses. Furthermore, the room was equipped with the Stanford *Emergency Manual*,[5] adapted for Massachusetts General Hospital as a cognitive aid, and a defibrillator. A code cart with simulated code medications including epinephrine, atropine, calcium chloride, and bicarbonate was readily available.

Regarding fire safety equipment, the actual fire alarm pull stations and fire extinguishers were labeled with a note to prevent the participants from accidentally initiating a response from police and security and the city fire department. Furthermore, an empty and appropriately labeled fire extinguisher was made available to the participants. An instructor was stationed in the hallway just outside of the in situ simulation OR to ensure safety protocols were maintained.

The debriefing room was separate from the in situ simulation OR and provided sufficient space and seating for the six participants

Copyright © 2019 Mai et al. This is an open-access publication distributed under the terms of the Creative Commons Attribution license.

and three facilitators to face each other. It was configured with an audiovisual setup that enabled all participants to see both camera views and the anesthesiology monitor. This room served as the introduction and orientation room, as well as the final debriefing room. Intraoperative and debriefing videos were recorded using B-Line Medical, and the videos recorded during the scenarios could be used in the debriefing to replay scenes from the simulation or for faculty-debriefer development.

Personnel

The learners consisted of a senior surgery resident paired with a surgical intern, as well as a senior anesthesia resident with a junior anesthesia resident or CRNA. Two OR nurses or one nurse and one surgical technologist were also recruited from the OR staff as learners for these interprofessional teams. If the participant team lacked a particular role, a faculty member assumed that role.

The implementation team for each session was an attending surgeon, an attending anesthesiologist, a nursing educator, and a simulation specialist. Matching instructor professions/disciplines to those of the participants ensured that the perspective of each participant was reflected during the debriefing.

Implementation

Each 90-minute session was divided into three parts: an orientation (10 minutes), the case with rapid cycle debriefing (65 minutes), and a final debriefing with course evaluations (15 minutes).

Prior to entering the OR, participants received a 10-minute orientation to ensure they understood the purpose of the simulation. Although the participants were unaware about the impending OR fire scenario, they were informed that the learning objectives of the simulation focused on a case scenario that addressed teamwork, communication skills, and crisis resource management. The participants were also informed about general issues such as the case background, teamwork objectives, equipment (including the vital sign monitor, code cart, IVs, medications, and record), simulation environment, and OR safety. Orientation slides used at our institution are provided in Appendix C and can be edited to fit other institutional setups. Participants also received a history and physical examination sheet and laboratory results (Appendix D), including a chemistry report, an ECG report, and an imaging report.

Faculty escorted participants into the simulated OR, where they assumed their respective roles (refer to Appendix A). The surgery participants scrubbed directly into the case, and the anesthesia participants started monitored anesthesia care. A World Health Organization Surgical Safety Checklist standard time-out was performed before proceeding with the case.

The first part of the scenario lasted approximately 10 minutes and ended when the learners identified the fire and formulated a management plan. The source of the fire was the electrosurgical unit igniting the oxygen from the patient's nasal cannula accumulating under the drapes. The scenario paused at this point for a 10-minute debriefing session in the simulation OR. After the debriefing, we allowed the learners to redo the first part of the scenario to practice the correct management of the OR fire, including extinguishing the contained fire. This practice session lasted an additional 5-10 minutes, and we again paused the session for a debriefing to focus on what the learners did differently compared to the first session.

After this second 5-minute debriefing, learners continued the simulation scenario. The fire had been extinguished and the drapes placed in a trash bag with the used alcohol prep sticks. A second fire ensued, and the learners were now expected to identify and manage an uncontained fire in the OR, which included an emergency evacuation of the OR team and patient into the adjacent hallway and shutting off all OR gases. Upon completion of this part, a 5-minute debriefing session occurred in the hallway to review indications for evacuation and how to safely evacuate patients to nearby areas with monitoring capabilities, including down stairwells.

At the conclusion of the case, we conducted a further 15-minute debriefing. A copy of our debriefing material is provided in Appendix E. This final debriefing session gave faculty the opportunity to review teamwork behaviors during a critical event and ways to manage this event. At the conclusion of the simulation activity, learners completed an anonymous evaluation form (Appendix F) prior to leaving the simulation space.

Assessment

The primary goal of this simulation was for learners to identify operations at elevated risk for an OR fire; manage the fire in terms of rescue, alert/alarm, contain, and extinguish/evacuate (RACE); and reduce adverse patient and team outcomes associated with OR fires. Within the extinguish/evacuate category, we emphasized how to utilize the fire extinguisher with the PASS mnemonic: pull pin/aim fire extinguisher nozzle/squeeze handle/sweep fire extinguisher nozzle back and forth over fire. Our assessment of the learners was formative and supportive, given the many limitations of the simulation scenarios, and was intended to help participants improve their

Copyright © 2019 Mai et al. This is an open-access publication distributed under the terms of the Creative Commons Attribution license.

performance. Of note, we did not assess participants' clinical skills and knowledge.

Confidentiality of the cases and individual performance was assured by instructors and participants alike. The debriefing process allowed learners to assess their prior knowledge, practice effective management, and identify alternative actions and behaviors.

Debriefing

We used the pause-and-discuss rapid cycle debrief technique, which gave learners the opportunity to reflect, debrief, and then redo the scenario. This technique allowed our participants to immediately correct and reinforce effective clinical and behavioral expectations and is described in more depth in the *MedEdPORTAL* publication "Rapid Cycle Deliberate Practice: Application to Neonatal Resuscitation."[6]

Two faculty conducted the debriefing, with the lead being taken by the less experienced debriefer to provide a faculty development opportunity. The debriefing first offered participants a chance to express any strong emotions they felt and to relieve stress. The lead debriefer then explained the facts of the case, reviewed the fire triangle (Figure), identified team members' roles in the fire triangle, and elucidated the correct actions/behaviors during a contained versus uncontained fire scenario (RACE).

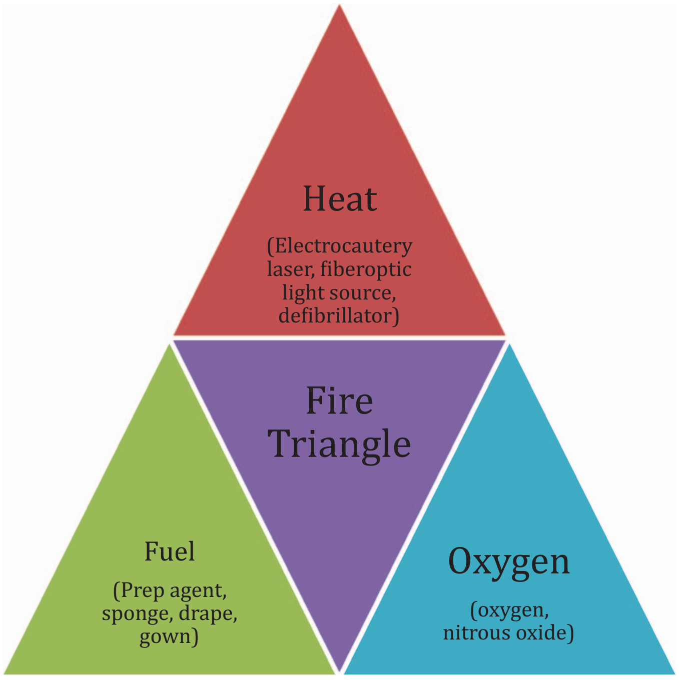

Figure. The fire triangle.

Other topics to focus on during the debriefing could include but are not limited to principles of crisis resource management, team dynamics, and communication skills. We concluded this debriefing by discussing examples of situations in which excellent team coordination required supportive behaviors from all team members.[7,8] Finally, the debriefing closed with faculty asking the learners to state their take-home messages and whether they would change anything if confronted with a similar situation in the OR environment. Learners assessed their own performance and gave feedback about the session using the anonymous evaluation form (Appendix F) on tablet computers before leaving the debriefing room.

Results

This simulation case was used from April to June 2015, with a total of 86 participants, including 23 surgical residents, 29 anesthetists (residents and CRNAs), 12 surgical technicians, 18 nurses, and four participants who declined to provide their role groups. The majority of learners agreed or strongly agreed that the simulation was applicable to their own practice (93%), that the simulation helped improve teamwork skills (80%), and that working in mixed teams was important to learning in this simulation exercise (93%). Furthermore, most learners agreed that they would change their clinical practice because of this simulation exercise (82%; see the Table).

A cursory qualitative thematic analysis of participants' comments indicated that participants were more aware of potential fire risks such as fuel sources from alcohol prep solution and redundant surgical drapes, heat sources from electrical cautery units causing hazards, and oxygen and nitrous oxide, which can potentiate the damage. They acknowledged the importance of identifying the locations of the fire extinguishers and alarms, how to use the appropriate extinguisher to manage the fire, and how to respond in the event of an OR fire. In addition, they appreciated the importance of effective teamwork, closed-loop communication, and speaking up during an OR fire crisis management event. With regard to the design and content of the simulation scenario itself, most agreed or strongly agreed that the session was realistic. However, some commented that more fake smoke (including adding a burning scent) in the scenario would create a sense of realism and urgency. In summary, participants found this case very useful, applicable to real practice, and likely to improve their teamwork skills.

Discussion

Intraoperative fire is an alarming and devastating event that can result in poor outcomes for both the care team and patient.

Copyright © 2019 Mai et al. This is an open-access publication distributed under the terms of the Creative Commons Attribution license.

Table. Percentage of Professional Groups Marking “Agree” or “Strongly Agree”

Question	Anesthetists (*n* = 29)	Nurses (*n* = 18)	Surgical Technicians (*n* = 12)	Surgeons (*n* = 23)	Not Specified (*n* = 4)	All Respondents (*N* = 86)
1. The simulation was realistic enough for me to engage in learning.	79%	89%	92%	70%	75%	80%
2. The simulation exercise helped me improve my teamwork skills.	83%	89%	100%	61%	75%	80%
3. The simulation exercise was clinically applicable to my practice.	97%	94%	100%	87%	75%	93%
4. I will change my practice as a result of this simulation exercise.	72%	83%	92%	87%	75%	82%
5. Working in mixed teams was important to my learning for this simulation exercise.	93%	94%	100%	91%	75%	93%

It is a rare occurrence in ORs, yet it places care providers, patients, and bystanders in extreme danger if the care team is not thoroughly prepared to identify and manage such a crisis. Our intraoperative team-training simulation involved an interprofessional team caring for a patient during an unexpected fire scenario. The following points were important aspects of this simulation.

First, case development involves an understanding of regulatory and compliance rules. The facility’s fire plans and responses should be researched so that the instructors follow the guidelines set by the institution. In addition, distinctions such as calling for help within the hospital versus calling for help from the outside (e.g., 911, fire department) need to be clear and easy, and practical guidelines should be distributed to participants.

Second, to appropriately conduct this scenario, specific safety measures need to be assessed and planned. Clear delineation between functional and nonfunctional equipment needs to be made, and any nonfunctional equipment must (1) be heavily labeled and (2) be removed from the area immediately following the simulation (and prior to initiating the final debriefing) if done in an in situ environment. Preventing the participants from activating the actual fire safety systems and processes is equally important. Another important aspect of this scenario development is the planning team. If the simulation session involves a multidisciplinary care team, the planning team must consist of the same role groups. It is vital that each discipline has responsibilities and actions to be completed. This is only possible when all perspectives are shared in the planning stages. This scenario did rely upon the expertise of individuals outside the perioperative arena. Involving facilities personnel and alerting local or hospital police and security to the simulation ensured cooperation across the institution.

Finally, the planning stage requires a decision on the method the session will follow. Employing the pause-and-discuss rapid cycle deliberate practice method allows the learners to immediately practice concepts discussed in the interim debriefings.[9] Moreover, creating realistic fire-related models requires a certain skill set that an experienced simulation specialist may provide. The simulation specialist needs to be acutely aware of the timing and responsibilities to reset the scenario while the debriefing is ongoing.

Some lessons learned during these sessions are to ensure confidentiality of the participants’ performance and to promote psychological safety among the learners. Many clinicians may not recall the skills and knowledge necessary to manage a fire in the OR, as they may not have had to access that information in a meaningful way. Reviewing the RACE algorithm and principles of fire, including the fire triangle, ensures a baseline knowledge in the context of emotional activation, given the simulation scenario. Communication specific to fire safety measures in the OR should be discussed during the debriefing. It is vitally important that clinicians in the immediate clinical environment are aware of the objectives of the simulation session and the proper methods of alerting others if an actual fire ensues during the training session.

An improvement to the scenario for the next iteration would be to make it more realistic. Based on our participants’ comments and suggestions, adding more fake smoke (including adding a burning scent) would create a sense of urgency in the case.

Limitations of this simulation session include the use of a general course evaluation to determine the course’s overall effectiveness. Ideally, hidden observation could be used to determine if this course ultimately changed participants’ behavior in the OR. Unfortunately, this is not feasible due to the costs required to hire observers, as well as a possible Hawthorne effect on participants’ behavior through the process of being observed. Anecdotally, we have heard from participants that there is a greater emphasis on OR fire mitigation and plans if an OR fire has occurred, although the actual rate of OR fires is too low to test these anecdotes in a systematic way.

Copyright © 2019 Mai et al. This is an open-access publication distributed under the terms of the Creative Commons Attribution license.

Appendices

A. Simulation Case Overview.docx

B. Teaching Points.docx

C. Slide Introduction.pptx

D. Surgical History and Physical Exam.docx

E. Debriefing Checklist.docx

F. Evaluation Form.docx

All appendices are peer reviewed as integral parts of the Original Publication.

Christine L. Mai, MD, MS-HPEd: Pediatric Anesthesiologist, Department of Anesthesia, Critical Care and Pain Medicine, Massachusetts General Hospital

Praelada Wongsirimeteekul, MD: Ophthalmologist, Department of Ophthalmology, Chiang Mai University

Emil Petrusa, PhD: Surgical Education Researcher, Department of Surgery, Massachusetts General Hospital

Rebecca Minehart, MD, MS-HPEd: Anesthesiologist, Department of Anesthesia, Critical Care and Pain Medicine, Massachusetts General Hospital

Maureen Hemingway, RN, DNP: Clinical Nurse Specialist, Massachusetts General Hospital

May Pian-Smith, MD: Anesthesiologist, Department of Anesthesia, Critical Care and Pain Medicine, Massachusetts General Hospital

Ersne Eromo, MD: Anesthesiologist, Department of Anesthesia, Critical Care and Pain Medicine, Massachusetts General Hospital

Roy Phitayakorn, MD, MHPE (Med): General and Endocrine Surgeon, Department of Surgery, Massachusetts General Hospital; Director of Medical Student Education, Department of Surgery, Massachusetts General Hospital; Director of Surgery Education Research, Department of Surgery, Massachusetts General Hospital; Senior Education Research and Development Consultant, New England Journal of Medicine Group

Disclosures
None to report.

Funding/Support
None to report.

Ethical Approval
Partners Human Research Committee at Massachusetts General Hospital, Partners Health Care, approved this study.

References

1. ECRI Institute announces new initiative to extinguish surgical fires. ECRI Institute website. https://www.ecri.org/press/Pages/Extinguish-Surgical-Fires.aspx. Published June 5, 2018.
2. Practice advisory for the prevention and management of operating room fires: an updated report by the American Society of Anesthesiologists Task Force on Operating Room Fires. *Anesthesiology*. 2013;118(2):271-290. https://doi.org/10.1097/ALN.0b013e31827773d2
3. DeMaria S Jr, Schwartz AD, Narine V, Yang S, Levine AI. Management of intraoperative airway fire. *Simul Healthc*. 2011;6(6):360-363. https://doi.org/10.1097/SIH.0b013e31821d420b
4. Konia MR, Jacobs X, Prielipp R, Reihsen T, Sweet R, Wangen K. Airway fire. *MedEdPORTAL*. 2012;8:9064. https://doi.org/10.15766/mep_2374-8265.9064
5. Stanford Anesthesia Cognitive Aid Group. *Emergency Manual: Cognitive Aids for Perioperative Critical Events 2016, V3.0*. Stanford, CA: Stanford Anesthesia Cognitive Aid Group; 2016.
6. Patricia K, Arnold J, Lemke D. Rapid cycle deliberate practice: application to neonatal resuscitation. *MedEdPORTAL*. 2017;13:10534. https://doi.org/10.15766/mep_2374-8265.10534
7. Stone JL, Aveling E-L, Frean M, et al. Effective leadership of surgical teams: a mixed methods study of surgeon behaviors and functions. *Ann Thorac Surg*. 2017;104(2):530-537. https://doi.org/10.1016/j.athoracsur.2017.01.021
8. Aveling E-L, Stone J, Sundt T, Wright C, Gino F, Singer S. Factors influencing team behaviors in surgery: a qualitative study to inform teamwork interventions. *Ann Thorac Surg*. 2018;106(1):115-120. https://doi.org/10.1016/j.athoracsur.2017.12.045
9. Eppich WJ, Hunt EA, Duval-Arnould JM, Siddall VJ, Cheng A. Structuring feedback and debriefing to achieve mastery learning goals. *Acad Med*. 2015;90(11):1501-1508. https://doi.org/10.1097/ACM.0000000000000934

Received: February 21, 2019
Accepted: September 7, 2019
Published: January 24, 2020

Copyright © 2019 Mai et al. This is an open-access publication distributed under the terms of the Creative Commons Attribution license.
