# Supplementary material for: Prevention and Management of Operating Room Fire: An Interprofessional Operating Room Team Simulation Case
Source: MedEdPORTAL. 2020 Jan 24;16:10871. doi: 10.15766/mep_2374-8265.10871 (PMC7012309; doi:10.15766/mep_2374-8265.10871)
Supplement: Supplementary file 1 — A. Simulation Case Overview.docx B. Teaching Points.docx C. Slide Introduction.pptx D. Surgical History and Physical Exam.docx E. Debriefing Checklist.docx F. Evaluation Form.docx [file mep-16-10871-s001.zip › A. Simulation Case Overview.docx]

| **SIMULATION CASE TITLE: Prevention and management of operating room fire** | |
| --- | --- |
| **PATIENT NAME:** Stanley Firestone  **PATIENT AGE:** 52-year-old man  **CHIEF COMPLAINT:** Enlarged lymph nodes in right neck | |
|  | |
| **Brief narrative** | Stanley Firestone is a 52-year old Caucasian man who presents with cervical LAD for 2.5 months.  He denies night sweats, weight loss, or any other type-B symptoms.  He has no history of head or neck malignancy including skin cancer.  A CT neck demonstrated significant LAD with necrotic centers at Level III on the right.  FNA and core biopsies of the lymph nodes were non-diagnostic.  The nodes are painful.  The pain is sharp and worse with pressure.  The patient has no other signs or symptoms related to the mass and denies any oral cancer history or significant abuse of tobacco/cigarettes.  The patient denies any recent weight loss and does not have any auto-immune diseases that he is aware of.  He also has no history of excessive bleeding with procedures or unexpected blood transfusions, but he is on ASA.‎  He has traveled to Mexico and Zambia, but not recently. He is scheduled for an excisional biopsy of a cervical lymph node under monitored anesthesia care (MAC).  Learners should be able to identify triggers of an OR fire and manage a contained as well as an uncontained fire in the OR with a multidisciplinary team. |
| **Primary Learning Objectives** | By the end of this session, the learners will be able to:   1. Identify situations conducive to OR fire 2. Manage OR fire in terms of Rescue, Alert/Alarm, Contain, Extinguish/Evacuate (RACE) 3. Reduce adverse outcomes associated with OR fires 4. Apply the core concepts of crisis management |
| **Critical Actions** | 1. First, participants should be able to detect an OR fire ignited from the electrocautery device and be able to manage the contained fire. Participants should be able to identify the Fire Triangle, and manage the OR fire according to the RACE algorithm. Review the PASS (Pull pin, aim the fire extinguisher, squeeze handle, sweep fire extinguisher) algorithm.  2. Next, participants are given a chance to redo the previously described contained OR fire scenario and practice and rehearse what they have learned.  3. Finally, participants have put out the contained OR fire and discussions about patient disposition occurs. However, an uncontained fire occurs when drapes that recently were on fire are accidentally placed in the trash over the alcohol prep sticks. Participants are expected to manage an uncontained OR fire according to the RACE algorithm. |
| **Learner Preparation** | Learners were given an introduction and oriented to the simulation environment (Appendix C) in a pre-brief. They are told, “The simulation is for your personal education and is in no way used for assessment or evaluation purposes. We follow the “Las Vegas Rule” in that everything that happens here stays here. Therefore, you cannot discuss this simulation session or anyone’s performance outside of your team today.” The history of previous illness, physical examination and laboratory investigation are presented to the participants at the beginning of the case. |

| Initial Presentation | | | |
| --- | --- | --- | --- |
| **Initial vital signs** | HR 83, BP 135/85, Temp 36.5, Pulse ox 97% (oxygen nasal canula), RR 12 | | |
| **Overall Appearance** | Patient is under sedation and prepped/draped  Patient already has a nasal canula oxygen and one PIV  Case already underway | | |
| **Participants and roles/Actors or confederates** | Participants and their roles:   - Surgery Intern - Surgery Senior - Anesthesia Junior - Anesthesia Senior - Nurse - Surgical technician   Confederates:   - OR nurse | | |
| **HPI** | Mr. Firestone is a 52-year old Caucasian man who presents with cervical LAD for 2.5 months.  He denies night sweats, weight loss, or any other type-B symptoms.  He has no history of head or neck malignancy including skin cancer.  A CT neck demonstrated significant LAD with necrotic centers at Level III on the right.  FNA and core biopsies of the lymph nodes were non-diagnostic.  The nodes are painful.  The pain is sharp and worse with pressure.  The patient has no other signs or symptoms related to the mass and denies any oral cancer history or significant abuse of tobacco/cigarettes.  The patient denies any recent weight loss and does not have any auto-immune diseases that he is aware of.  He also has no history of excessive bleeding with procedures or unexpected blood transfusions, but he is on ASA.‎  ‎‎  SOCHX: He has traveled to Mexico and Zambia, but not recently. Former tobacco abuse, quit almost 30 years ago. Occasional ETOH use. Exercises regularly.  ROS:  Constitutional: Denies fever, chills, night sweats, weight loss, and anorexia. Positive for fatigue.  HEENT: No hx of hoarseness, epistaxis, mouth sores, swollen eyes, blurred vision, exophthalmos, or double vision.  CV: Denies SOB or distal swelling. No palpitations or syncope.  PULM: Denies hemoptysis, asthma, or pleuritic chest pain. Positive chronic cough.  GI: Denies nausea, abdominal pain, GERD, or diarrhea. No jaundice. Occasional constipation.  GU: No urinary incontinence or urinary tract infections.  MS: Minimal arthritis-type pain.  DERM: Denies any skin rash or pruritus or dry skin.  NEURO: Denies weakness, tremors, or problems with gait. Frequent headaches.  PSYCH: No depression or anxiety.  HEME: As above. No bleeding hx. Denies any lymph node enlargement. | | |
| Past Medical/Surgical History | Medications | Allergies | Family History |
| PMEDHX: Pulmonary embolism after air travel treated previously in 2010.  Chronic lung disease on 2L nasal canula at night  Chronic cough  PSURGHX: none | Aspirin (ACETYLSALICYCLIC Acid (ASPIRIN)) 81MG (81 MG TABLET Take 1) PO QD  Flonase (FLUTICASONE Nasal Spray) 1-2 SPRAY NAS QD; No change (Taking)  Albuterol inhaler (2 puffs) PRN with exercise | NKA | Denies history of malignancy in family, thyroglossal duct cyst, endocrinopathies, or connective tissue disorders |
| Physical Examination | | | |
| General | Well-developed man in no distress | | |
| HEENT | The most prominent lymph node is located in the Level III-IV region of the RIGHT neck. It measures approximately 2 cm in size. On palpation it is firm and non-palsatile. It is fixed to the surrounding structures. It is painful to deep palpation and there are no signs of infection. | | |
| Neck | The most prominent lymph node is located in the Level III-IV region of the RIGHT neck. It measures approximately 2 cm in size. On palpation it is firm and non-palsatile. It is fixed to the surrounding structures. It is painful to deep palpation and there are no signs of infection. The mass does not interfere with his neck movement. | | |
| Lungs | CTA B | | |
| Cardiovascular | Nl S1S2 | | |
| Abdomen | Soft, NT/ND with +BS. No inguinal or axillary LAD. | | |
| Neurological | Normal consciousness, no neurological deficit | | |
| Skin | No other masses or lesions noted. | | |
| GU | WNL | | |
| Psychiatric | Normal MSE | | |

| Instructor Notes - Changes and CASE Branch Points |
| --- |

| Time |  | Key Points to make |
| --- | --- | --- |
| Start | Orient Team to OR | Patient is under sedation and prepped/draped  Patient already has a nasal oxygen canula, one PIV  Case already underway  Vitals: HR 83, BP 135/85, Temp 36.5, Pulse ox 97% (oxygen nasal canula), RR 12 |
| Minute 0-5 | Case starts | JCAHO time-out  Surgery resident inject local anesthesia  Surgery resident makes incision  Surgery resident cauterize the wound  Smoke occurs underneath the drapes from dried ice to simulate OR fire  When fire occurs vitals change: HR 115 BP 140/90 Pulse ox 92% RR 12  Ideally, the surgery resident or scrub tech yells “Fire” |
| Minute 5 | Case ends | Once participants have either ask for burn sugeon or decide to continue procedure, case ends and debriefing starts. |
| Minute 5-10 | DEBRIEF: Reactions, Fire Triangle reviewed, Team members role in Fire Triangle identified, contained OR fire management according to the RACE algorithm, crisis resource management and team dynamics reviewed with advocacy inquiry  Tell participants to stay gloved/gowned during debrief | |
| Minute 10-20 | Case starts over | Tell the participants that they will now have an opportunity to redo the contained OR fire case scenario. Participants have just done the JCAHO time-out, injected local, and made the incision. The surgery resident has just used the electrocautery.  Participants expected to recognize the contained OR fire, and manage the fire using the RACE or PASS algorithm Use advocacy inquiry to engage the particpants in what they did differently, review RACE and PASS components. |
|  |  | Tell them:   - You all are going to have an opportunity to redo the case. You have just performed the JCAHO time-out, the surgical site was injected with local anesthetic and incision has occurred. The electrocautery is being used. |
| Minute 20-25 | DEBRIEF: Reaction, use advocacy inquiry to engage the participants in what they did differently, review Fire Triangle, RACE and PASS algorithms.  Tell participants to stay gloved/gowned during debrief | |
| Minute 25-35 | Case continues | Ask them:   - You have now put out the fire successfully, what do you want to do with the patient? Cancel case/continue? MAC versus general anesthesia? |
|  |  | Case continues, but the drapes have been pulled off while on fire and accidentally thrown on the trash bag that contained the alcohol prep sticks. A projection image of a fire is shown on the OR wall.  Vitals: HR 95 BP 145/95 Pulse ox 95% RR 15  Participants expected to recognize the uncontained OR fire, evacuate themselves and their patient into the hallway. |
| Minute 35-40 | DEBRIEF: Reactions, management of uncontained OR fire using RACE algorithm. Evacuation of the participants and the patient into the hallway. Identify and turn off the oxygen shut off valve in the hallway. Use advocacy inquiry to review crisis resource management and team dynamics.  Participants can now take off gloves/gowns during debrief | |
| Minute 40-55/60 | Move group to debriefing room | Review the etiology of the case and the management of contained versus uncontained fire based on the RACE algorithm and PASS components. |
|  | Wrap-up and evaluation. | |

**Ideal Scenario Flow**

In this scenario, beginning with the case, the learners enter the OR to find an adult patient under sedation and prepped/draped. Patient already has nasal canula oxygen with PIV. Case is about to begin with a JCAHO time-out. Surgeon participants inject local anesthetic and make incision. While surgeon use the electrocautery on the wound, a fire occurs underneath the drapes and smoke can be identified. At this point, someone on the team should identify the smoke and yell out “Fire!” The team should pull the fire alarm, call the correct phone number to alert the CODE RED, and put out the fire. Team delegation of tasks and closed loop communication should be performed. Once the team has identified the fire and the smoke is extinguished using saline on the field, case ends and debriefing starts in the OR with a review of the Fire Triangle, RACE algorithm, and PASS components.

Following the debrief participants will redo the case. The team has just completed a JCAHO time-out. The surgeons inject local anesthesia, make an incison and use the electrocautery. A fire ensues and the participants will once again manage the contained OR fire following the RACE algorithm. The scenario is paused for an in-OR debriefing that follows advocacy inquiry to engage the participants in what they did and might do differently. An instructor reviews the Fire Triangle, RACE algorithm in terms of contained OR fire, and PASS components.

Following this debrief, the case resumes with surgeons and the anesthesia team discussing about post-operative disposition for this patient. The drapes have been removed and are placed on top of a trash bin that has alcohol prep sticks. An uncontained fire ensues in the trash bin and the participants will have to identify the fire and work together to evacuate themselves and the patient from the operating room. The scenario ends with another debrief about participant reactions, review of the Fire Triangle, evacuation strategies, RACE algorithm in terms of uncontained OR fire, and identification of the location of the oxygen shut off valve. We used advocacy inquiry to review crisis resource management and team dynamics.

**Anticipated Management Mistakes**

For this OR fire case scenario, anticipated management mistakes include:

1. Participants may be unfamiliar with the Fire Triangle. Even though we are expected to review fire safety annually in our institution, some participants may be unfamiliar with the triggers of an OR fire. We anticipated this and had a focused debrief to review the components of the Fire Triangle.
2. Team expected to know the RACE algorithm. Even though we are expected to review the RACE algorithm annually in our fire safety modules, this review usually is in theory and not practiced in real-time. This case scenario presents two instances where the RACE algorithm can be practiced for a contained fire as well as an uncontained fire situation.
3. Team may be unfamiliar with the evacuation and exit strategies. During the debriefing, we review vertical and horization evacuation strategies.
4. The team members are expected to delegate roles and tasks. In an environmental emergency such as an OR fire, there may not be enough staff immediately available. The team members in the OR should discuss about task delegation and role assignments in anticipation of an immediate evacuation scenario.
