# Supplementary material for: Prevention and Management of Operating Room Fire: An Interprofessional Operating Room Team Simulation Case
Source: MedEdPORTAL. 2020 Jan 24;16:10871. doi: 10.15766/mep_2374-8265.10871 (PMC7012309; doi:10.15766/mep_2374-8265.10871)
Supplement: Supplementary file 1 — A. Simulation Case Overview.docx B. Teaching Points.docx C. Slide Introduction.pptx D. Surgical History and Physical Exam.docx E. Debriefing Checklist.docx F. Evaluation Form.docx [file mep-16-10871-s001.zip › B. Teaching Points.docx]

**RACE**

CORRECT ACTIONS/BEHAVIORS

|  | **Contained Fire** | **Uncontained Fire** |
| --- | --- | --- |
| **Rescue:**  **Patient & Team** | **Surgeon/Scub Nurse/Technician:** remove drapes, place water/saline on wound and drapes to prevent fire spread  **Anesthesia:** shut off oxygen and remove nasal cannula | Same as with contained fires, but faster and will move patient onto a stretcher and into the hallway |
| **Alert/Alarm** | **Circulator:** find fire extinguisher, contact the operating room desk, consider pulling fire alarm in hallway | Same as with contained fires, but **circulator** should delegate task of pulling fire alarm to someone in the hallway and follow local institution’s policies or can call security |
| **Contain/Confine** | - Ensure patient is no longer on fire - Isolate all flammable materials so fire cannot spread throughout the room - Consider placing someone at the door to prevent other staff from rushing into the room and potentially exposing themselves to the fire | Prevent other staff from rushing into the room and potentially exposing themselves to the fire.  Turn off oxygen valves outside of the room. |
| **Extinguish or Evacuate** | Remind team that alcohol fires can be very difficult to see initially. If fire still going, someone must use the fire extinguisher (PASS)  **P** – Pull pin  **A** – Aim fire extinguisher nozzle  **S** – Squeeze handle  **S** – Sweep fire extinguisher nozzle back and forth over the fire | - Can try to extinguish fire, but someone must make decision to evacuate the OR. - Anesthesia residents will grab Ambu bag and some drugs and unlock OR table - Surgery residents will place moist towel/gauze over wound - Scrub will move table to opposite wall and clear a path to the door - Entire team must work together to move patient into hallway |

Note: if it is a chemical fire extinguisher, it cannot be used on an open wound as the chemicals are toxic. Discuss different types of fire extinguishers.

**IMPORTANT:**  RACE is an iterative cycle and they should go back through the steps to reassess. For example, now that the patient is in the hallway, what are they going to do?

| **R** | Keep the team safe and make sure no further staff go into the room |
| --- | --- |
| **A** | Ensure that the fire department is coming |
| **C** | Make sure the doors to the OR are closed so that the fire is somewhat contained/compartmentalized. Shut off gas to correct operating room |
| **E** | Be prepared to go into another OR or move the patient past the fire doors |

**OR FIRE TRIANGLE**

**Key Point:** Most OR Fires can be prevented if the OR team understood the nature of the hazards and how to minimize the risks. Everyone should maintain awareness of these risks and ensure excellent pre- and intra-operative communication.

|  | **FUEL (nursing)** | **HEAT (surgery)** | **OXIDIZER (anesthesia)** |
| --- | --- | --- | --- |
| Components of the fire triangle | Preparatory Agents  Swabs/Sponges  Drapes/towels  Gowns  Gastrointestinal Gases  Patient’s Hair | Electrocautery  Laser  Fiberoptic light source  Defibrillators | Oxygen  Nitrous Oxide |
| Preventative Actions: | 1. Moisten all swabs and sponges before passing them to surgeons 2. Avoid alcohol as the primary skin prep solution 3. Label all solutions in the sterile field 4. Wet patient’s hair with aqueous gel | 1. Let preparatory solution dry completely before draping 2. Use electrocautery or laser sparingly 3. Holster electrosurgical unit properly when not in use 4. Keep tip of electrosurgical unit as clean as possible 5. Place lasers in stand-by mode when not in use 6. Never lay fiberoptic or headlight source on surgical drapes 7. Open all lumens using cold cutting (scissors/scalpel) | 1. Use minimal oxygen concentration 2. Use medical air when possible because it has less than 30% oxygen 3. Avoid air leaks from anesthesia circuit 4. Don’t allow oxygen to collect beneath drapes 5. Know oxygen shut off locations |
