# Supplementary material for: Prevention and Management of Operating Room Fire: An Interprofessional Operating Room Team Simulation Case
Source: MedEdPORTAL. 2020 Jan 24;16:10871. doi: 10.15766/mep_2374-8265.10871 (PMC7012309; doi:10.15766/mep_2374-8265.10871)
Supplement: Supplementary file 1 — A. Simulation Case Overview.docx B. Teaching Points.docx C. Slide Introduction.pptx D. Surgical History and Physical Exam.docx E. Debriefing Checklist.docx F. Evaluation Form.docx [file mep-16-10871-s001.zip › C. Slide Introduction.pptx]

## Slide 1
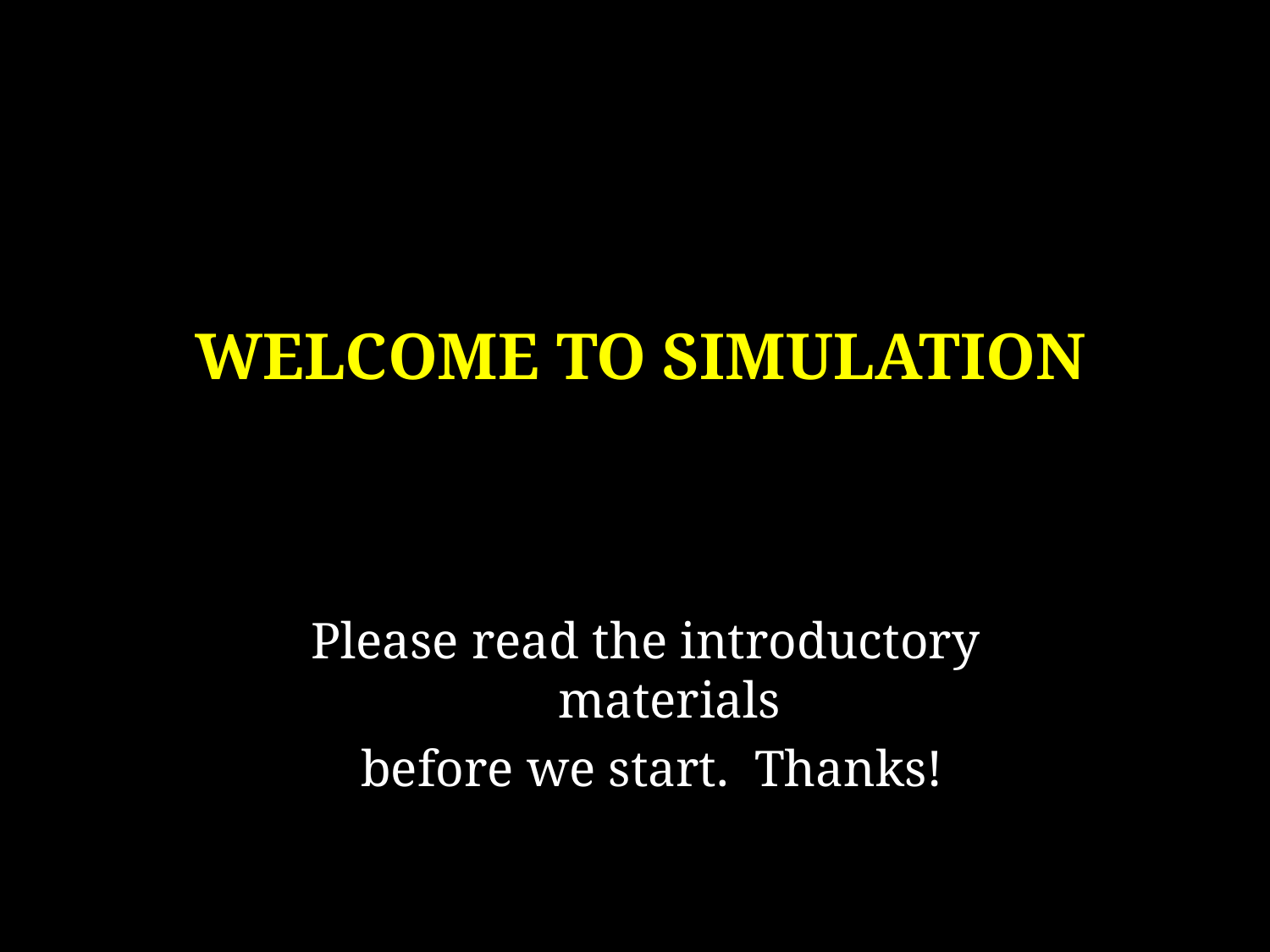

# WELCOME TO SIMULATION
Please read the introductory materials
 before we start. Thanks!

## Slide 2
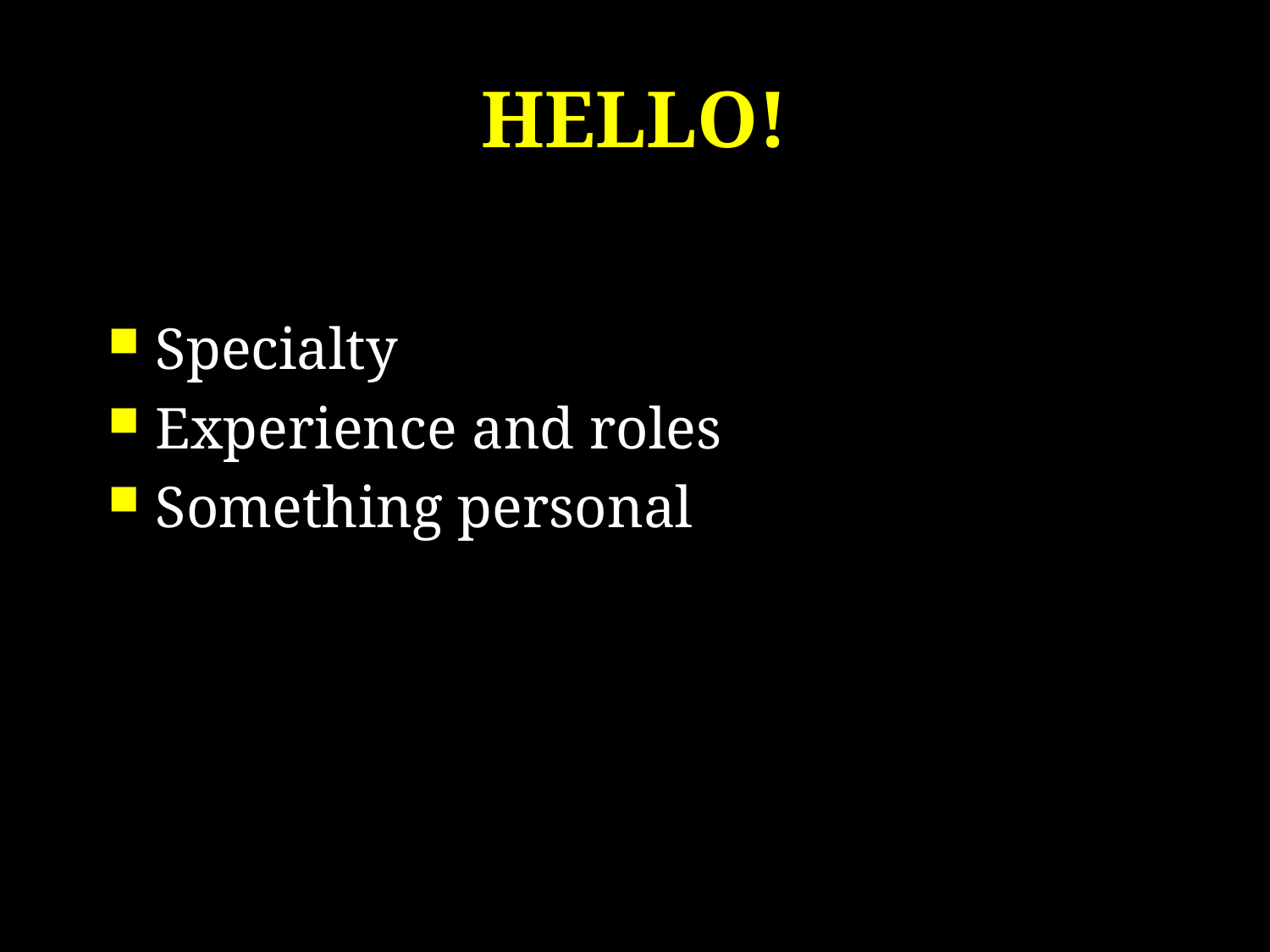

# HELLO!
Specialty
Experience and roles
Something personal

## Slide 3
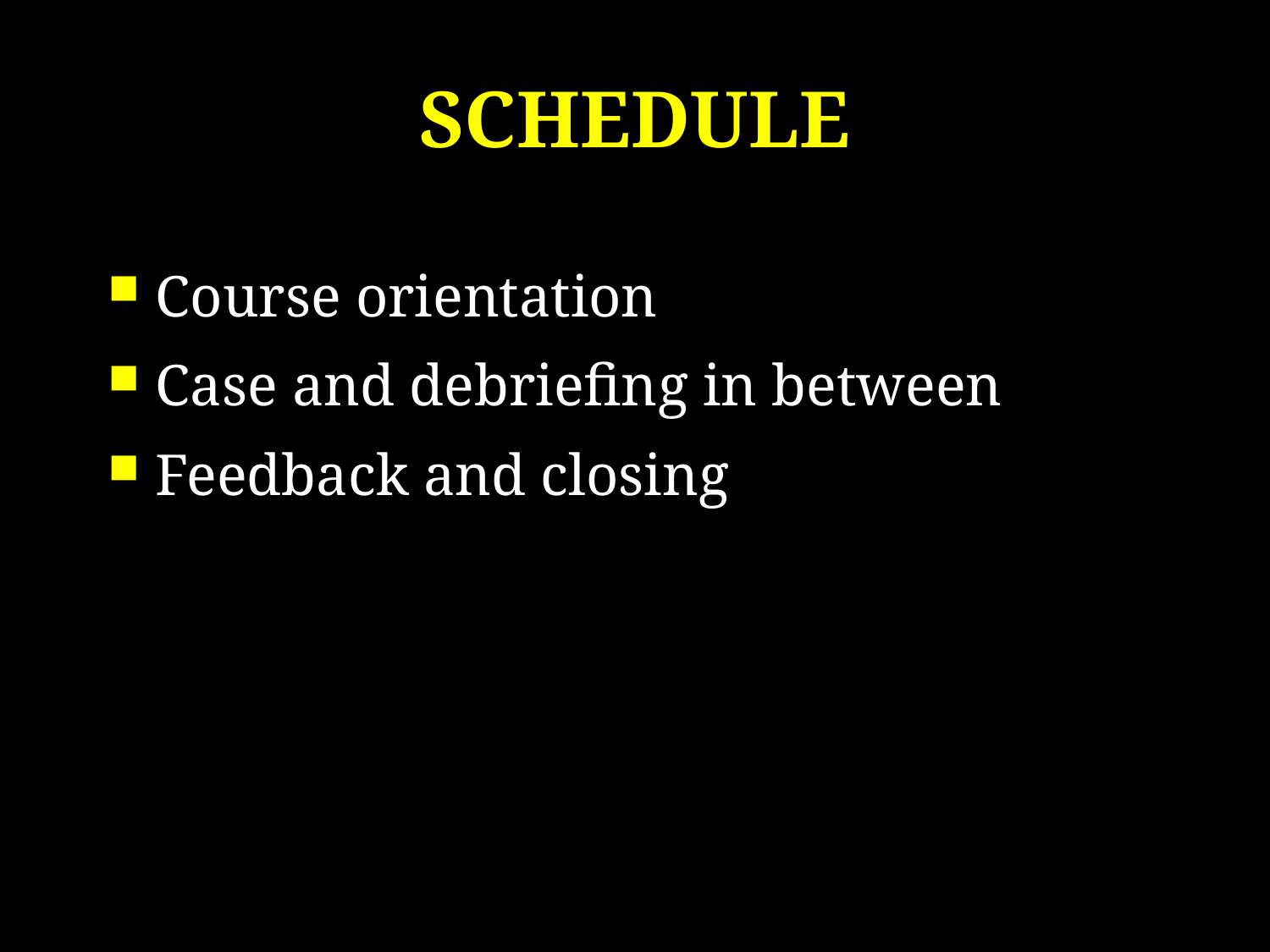

# SCHEDULE
Course orientation
Case and debriefing in between
Feedback and closing

## Slide 4
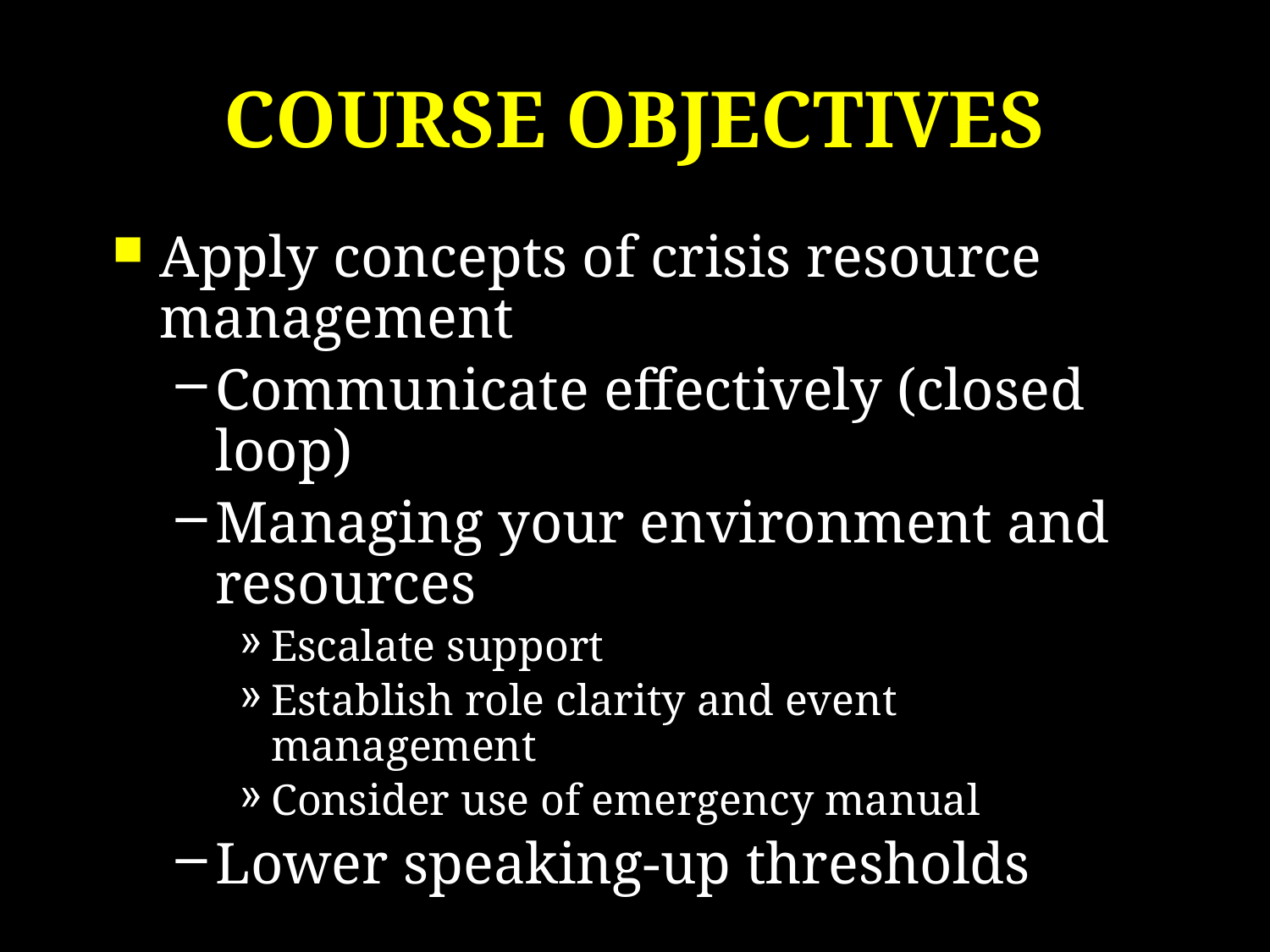

# COURSE OBJECTIVES
Apply concepts of crisis resource management
Communicate effectively (closed loop)
Managing your environment and resources
Escalate support
Establish role clarity and event management
Consider use of emergency manual
Lower speaking-up thresholds

## Slide 5
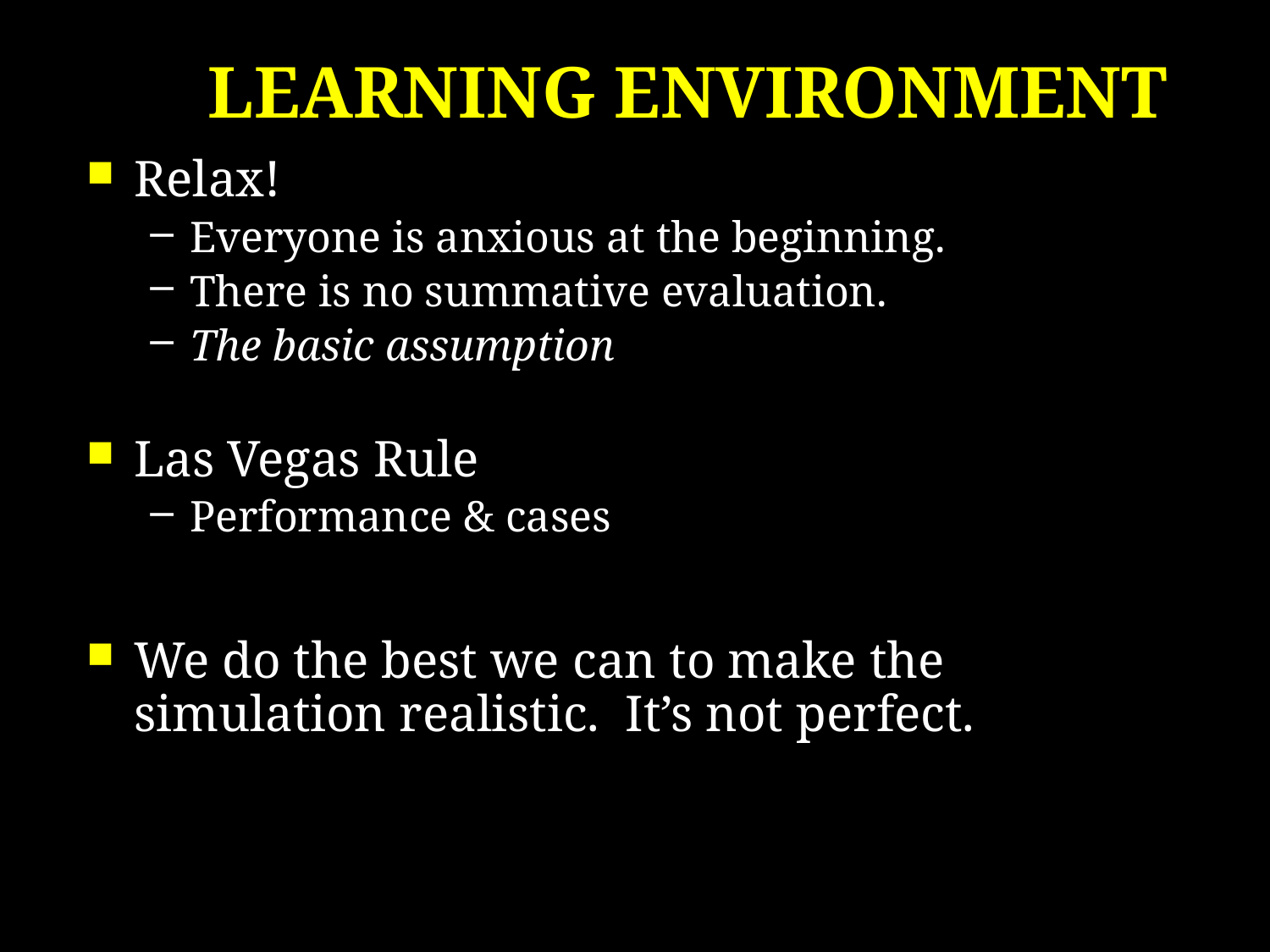

# LEARNING ENVIRONMENT
Relax!
Everyone is anxious at the beginning.
There is no summative evaluation.
The basic assumption
Las Vegas Rule
Performance & cases
We do the best we can to make the simulation realistic. It’s not perfect.

## Slide 6
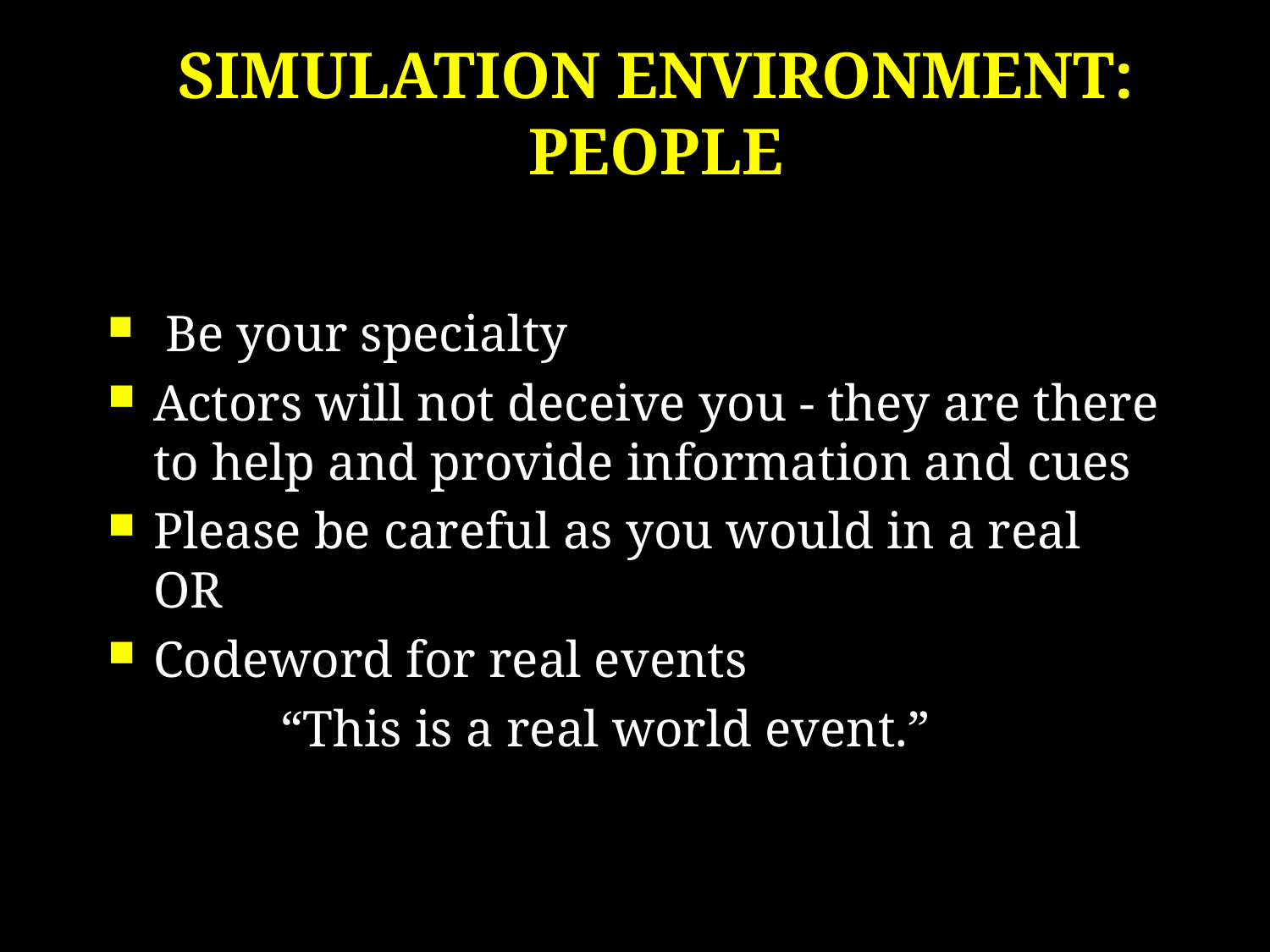

SIMULATION ENVIRONMENT:
PEOPLE
 Be your specialty
Actors will not deceive you - they are there to help and provide information and cues
Please be careful as you would in a real OR
Codeword for real events
		“This is a real world event.”

## Slide 7
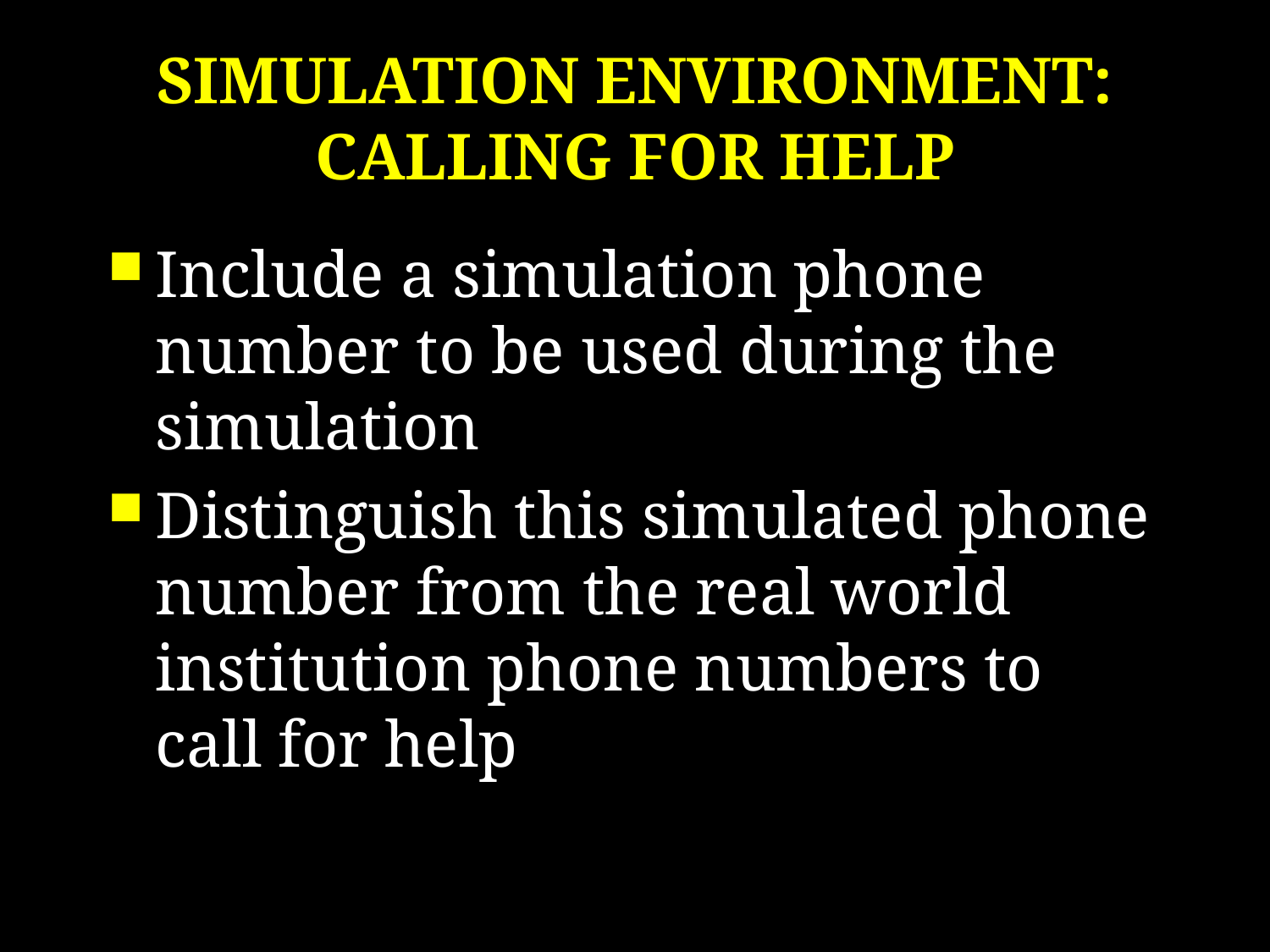

# SIMULATION ENVIRONMENT: CALLING FOR HELP
Include a simulation phone number to be used during the simulation
Distinguish this simulated phone number from the real world institution phone numbers to call for help

## Slide 8
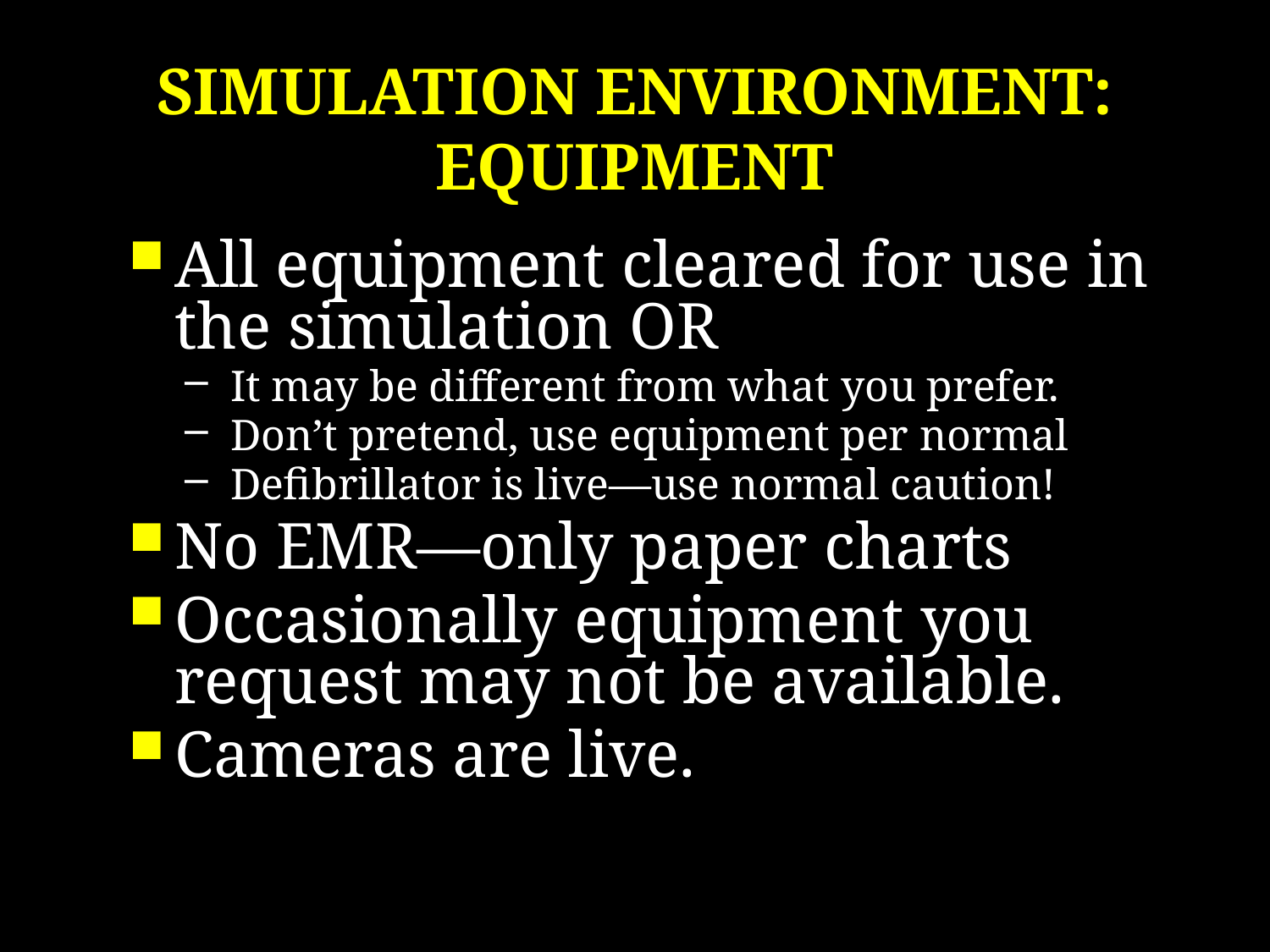

# SIMULATION ENVIRONMENT: EQUIPMENT
All equipment cleared for use in the simulation OR
It may be different from what you prefer.
Don’t pretend, use equipment per normal
Defibrillator is live—use normal caution!
No EMR—only paper charts
Occasionally equipment you request may not be available.
Cameras are live.

## Slide 9
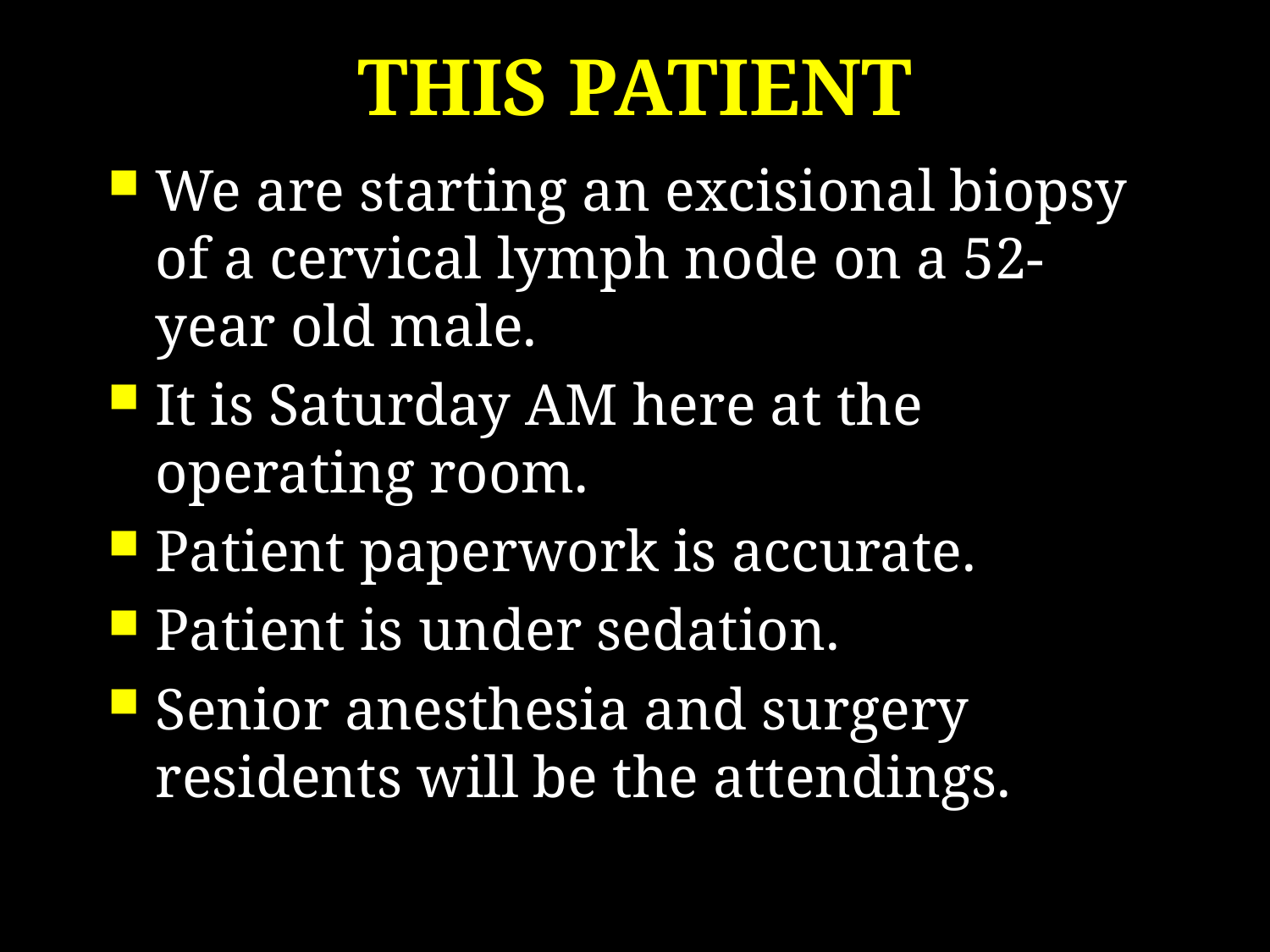

# THIS PATIENT
We are starting an excisional biopsy of a cervical lymph node on a 52-year old male.
It is Saturday AM here at the operating room.
Patient paperwork is accurate.
Patient is under sedation.
Senior anesthesia and surgery residents will be the attendings.

## Slide 10
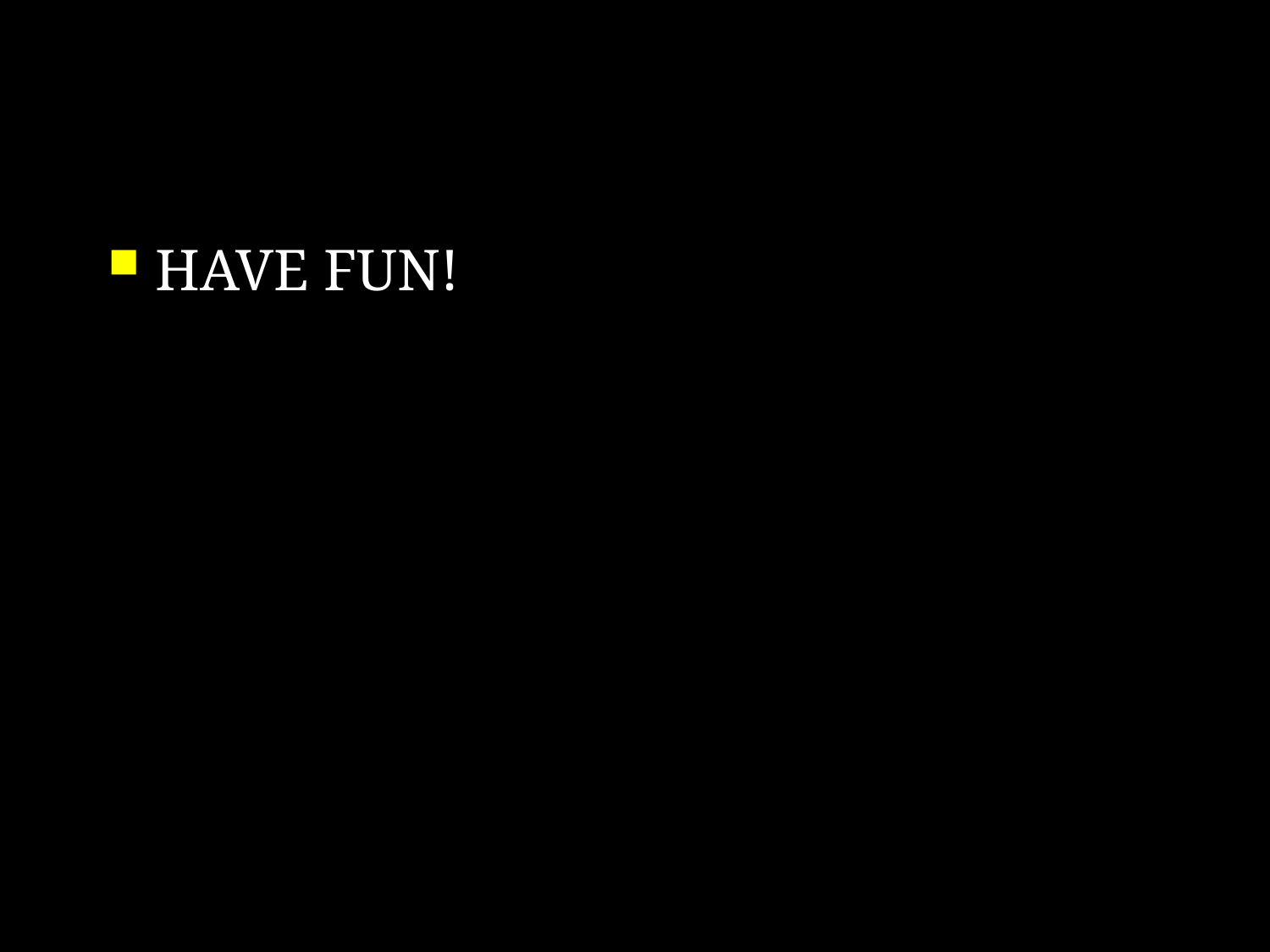

#
HAVE FUN!
