# Supplementary material for: Prevention and Management of Operating Room Fire: An Interprofessional Operating Room Team Simulation Case
Source: MedEdPORTAL. 2020 Jan 24;16:10871. doi: 10.15766/mep_2374-8265.10871 (PMC7012309; doi:10.15766/mep_2374-8265.10871)
Supplement: Supplementary file 1 — A. Simulation Case Overview.docx B. Teaching Points.docx C. Slide Introduction.pptx D. Surgical History and Physical Exam.docx E. Debriefing Checklist.docx F. Evaluation Form.docx [file mep-16-10871-s001.zip › D. Surgical History and Physical Exam.docx]

Top of Form

‎

**NAME:** Firestone, Stanley **Sex:** M

**MRN:**  336 17 14

**DOB:**  02-06-1963

**HISTORY AND PHYSICAL**‎

**CC:**  Neck mass‎

‎‎

**HPI:**  Mr. Firestone is a 52-year old Caucasian man who presents with cervical LAD for 2.5 months.  He denies night sweats, weight loss, or any other type-B symptoms.  He has no history of head or neck malignancy including skin cancer.  A CT neck demonstrated significant LAD with necrotic centers at Level III on the right.  FNA and core biopsies of the lymph nodes were non-diagnostic.  The nodes are painful.  The pain is sharp and worse with pressure.  The patient has no other signs or symptoms related to the mass and denies any oral cancer history or significant abuse of tobacco/cigarettes.  The patient denies any recent weight loss and does not have any auto-immune diseases that he is aware of.  He also has no history of excessive bleeding with procedures or unexpected blood transfusions, but he is on ASA.‎  He has traveled to Mexico and Zambia, but not recently.

‎‎

**PMEDHX:‎**

Pulmonary embolism after air travel treated previously in 2010

Obstructive lung disease on 2L N/C at night

Cough

‎‎‎

**PSURGHX:‎**

None

‎‎‎

**MEDS:‎**

Aspirin (ACETYLSALICYLIC Acid (ASPIRIN)) 81 MG (81 MG TABLET Take 1) PO QD Flonase (FLUTICASONE Nasal Spray) 1-2 SPRAY NAS QD; No Change (Taking)

Albuterol inhaler (2 puffs) PRN with exercise

‎‎‎

**ALL:‎**  NKA

‎

‎‎**FAMHX**:  N/C;  No family history of neck malignancy, thyroglossal duct cyst, endocrinopathies, or connective tissue disorders

‎

**SOCHX**:‎  Former tobacco abuse, quit almost 30 years ago.  Occasional ETOH use.  Exercises regularly.

‎‎

**ROS:‎**

‎‎Constitutional:  Denies fever, chills, night sweats, weight loss or anorexia.‎ ‎Positive for fatigue.

HEENT:  No hx of hoarseness, epistaxis, mouth sores, swollen eyes, blurred vision, exophthalmos, or double vision. ‎‎

CV:  Denies SOB, or distal swelling.  No chest pain or palpitations or syncope.  ‎

PULM:  Denies hemoptysis, asthma, or pleuritic chest pain.  ‎‎Chronic cough.

GI:  Denies nausea, abdominal pain, GERD, or diarrhea.  No jaundice.  Occasional constipation.

GU:  No urinary incontinence or urinary tract infections.‎‎

MS:  Minimal arthritis-type pain.‎

DERM:  Denies any skin rash or pruritus or dry skin.‎‎

NEURO:  Denies weakness, tremors, or problems with gait.‎‎  Frequent headaches.

PSYCH:  No depression or anxiety.  ‎

HEME:  Denies any lymph node enlargement‎ ‎

‎‎

**VITAL SIGNS**:‎  BP 119/77, P 68, T 37.8, Ht 71 in, Wt 198.2 lb, O2 SAT 95-97 on 2L N/C, BMI 27.6, PAIN LEVEL (0-10) 3

‎GEN:  Well-developed man in no distress‎

HEENT:  The most prominent lymph node is located in the Level III-IV region of the RIGHT neck.  It measures approximately 2 cm in size.  On palpation it is firm and non-pulsatile.  It is fixed to the surrounding structures.  It is painful to deep palpation and there are no signs of infection.‎

CV:  Nl S1S2‎

PULM:  CTA B‎

ABD:  Soft, NT/ND with +BS‎.  No inguinal or axillary LAD.

EXTR:  No edema

SKIN:  No other masses or lesions noted

MS:  The mass does not interfere with movement of his neck

‎‎

**LABS:**  I reviewed his labs over the last year and find no increased risk of bleeding

‎

**IMAGING:**  See CAS for prior imaging results.  I reviewed the images and agree with the findings.‎

I performed a bedside ultrasound examination and found that the mass is SOLID with a necrotic center and has increased vascularity.  There are no aberrant blood vessels in the vicinity of the mass.‎ It is well away from the carotid, external and internal jugular vein.

                      ‎

**IMPRESSION:**  LAD of unspecified nature. ‎The differential diagnosis includes neoplastic disease including primary lymphoma or metastatic malignancy.  It is also possibly infectious or reactive.

‎‎

**PLAN:**  The patient would like the mass to be excised for full pathology work-up.  Half of the lymph node should be sent for frozen section for lymphoma work-up, the other half will be sent for ID work-up (mycobacterium stain and culture, gram stain and culture, fungal stain and culture). We discussed the risks and benefits of the procedure as well as possible damage to critical structures in the neck. All questions answered to his satisfaction.

‎‎

‎‎‎

Electronic Signature __________________________________________‎

ADDENDUM: Based on the discussions in PATA, we will proceed with MAC with general anesthesia as back-up to minimize post-operative respiratory complications. He is in full agreement with this plan. All questions answered.
