# Supplementary material for: Prevention and Management of Operating Room Fire: An Interprofessional Operating Room Team Simulation Case
Source: MedEdPORTAL. 2020 Jan 24;16:10871. doi: 10.15766/mep_2374-8265.10871 (PMC7012309; doi:10.15766/mep_2374-8265.10871)
Supplement: Supplementary file 1 — A. Simulation Case Overview.docx B. Teaching Points.docx C. Slide Introduction.pptx D. Surgical History and Physical Exam.docx E. Debriefing Checklist.docx F. Evaluation Form.docx [file mep-16-10871-s001.zip › E. Debriefing Checklist.docx]

**Debriefing Check List**

|  |
| --- |
| **After First Fire (Nasal Cannula Fire)**   - Reactions - Fire Triangle Components: Introduce - How do the different team members contribute to the fire triangle? - RACE (Rescue/Alert/Contain/Extinguish/Evacuate) in terms of contained OR fire - PASS (Pull/Aim/Squeeze/Sweep)   **After Repeat of First Fire (Nasal Cannula Fire)**   - Use advocacy inquiry^*^ to engage the participants in what they did differently - Review RACE or PASS components - Triangle: OR sources of Fire (light sources and lasers) - Preventive Actions   **After Second Fire (Drape Fire)**   - Reactions - RACE in terms of uncontained OR fire   - OR O2 shut off valve locations   - Vertical vs horizontal OR evacuations   **Group Debrief**   - Following and leading with event manager - Trust in leader and necessity to have someone listening |

* Rudolph JW, et al. There’s no such thing as “nonjudgmental” debriefing: a theory and method for debriefing with good judgment. Simul Healthc. 2006; 1: 23-25.
