# Supplementary material for: Prevention and Management of Operating Room Fire: An Interprofessional Operating Room Team Simulation Case
Source: MedEdPORTAL. 2020 Jan 24;16:10871. doi: 10.15766/mep_2374-8265.10871 (PMC7012309; doi:10.15766/mep_2374-8265.10871)
Supplement: Supplementary file 1 — A. Simulation Case Overview.docx B. Teaching Points.docx C. Slide Introduction.pptx D. Surgical History and Physical Exam.docx E. Debriefing Checklist.docx F. Evaluation Form.docx [file mep-16-10871-s001.zip › F. Evaluation Form.docx]

**PROGRAM EVALUATION**

Thank you for participating in our OR simulation! Your feedback is extremely helpful in allowing us to adapt the program to best benefit our staff.

Date of Simulation Exercise: ________________

Name (optional): _____________________________________________

OR Role (optional):

☐ Circulating RN, ☐ Anesthesia, ☐ Surgeon, ☐ Other ______________

**1. The simulation was realistic enough for me to engage in learning.**

**Strongly** Somewhat Somewhat **Strongly**

**Disagree** Disagree Disagree **Neutral** Agree Agree **Agree**

1 2 3 4 5 6 7

Comments:_______________________________________________________________________________________________________________________

**2. The simulation exercise helped me improve my teamwork skills.**

**Strongly** Somewhat Somewhat **Strongly**

**Disagree** Disagree Disagree **Neutral** Agree Agree **Agree**

1 2 3 4 5 6 7

If agree, what skills were improved?:___________________________________ ________________________________________________________________

**3. The simulation exercise was clinically applicable to my practice.**

**Strongly** Somewhat Somewhat **Strongly**

**Disagree** Disagree Disagree **Neutral** Agree Agree **Agree**

1 2 3 4 5 6 7

Comments:_______________________________________________________________________________________________________________________

**4. I will change my practice as a result of this simulation exercise.**

**Strongly** Somewhat Somewhat **Strongly**

**Disagree** Disagree Disagree **Neutral** Agree Agree **Agree**

1 2 3 4 5 6 7

If agree, what change(s) will be made?:__________________________________________________________________________________________________________________________

**5. Working in mixed teams was important to my learning for this simulation exercise.**

**Strongly** Somewhat Somewhat **Strongly**

**Disagree** Disagree Disagree **Neutral** Agree Agree **Agree**

1 2 3 4 5 6 7

Comments:_______________________________________________________________________________________________________________________

**6. Please make any comments that will help us improve this program:** ________________________________________________________________________________________________________________________________________________________________________________________________

________________________________________________________________

________________________________________________________________

________________________________________________________________

________________________________________________________________________________________________________________________________________________________________________________________________

________________________________________________________________

________________________________________________________________
